# Combined Effects of Atorvastatin and Glucose Deprivation on Metabolic Stress and Lipid-Raft Disruption in Glioblastoma and Breast Cancer Cells

**DOI:** 10.3390/pharmaceutics17101275

**Published:** 2025-09-29

**Authors:** Walhan Alshaer, Yousef Ijjeh, Nowar Alsarayreh, Dana A. Alqudah, Alaa Rifai, Ahmed Abu-Siniyeh, Mohammad Alsalem

**Affiliations:** 1 Cell Therapy Center, The University of Jordan, Amman 11942, Jordan; yousef.a.ijjeh@gmail.com (Y.I.); nowarsarayra@gmail.com (N.A.); d_alqudah@ju.edu.jo (D.A.A.); refai.ala278@gmail.com (A.R.); 2 Department of Medical Laboratory Sciences, Faculty of Science, The University of Jordan, Amman 11942, Jordan; a.siniyeh@ju.edu.jo; 3 Department of Anatomy and Histology, School of Medicine, The University of Jordan, Amman 11942, Jordan; m_alsalem@ju.edu.jo

**Keywords:** atorvastatin, glucose starvation, cancer, combination therapy, lipid rafts

## Abstract

**Background/Objectives:** Atorvastatin, a lipophilic HMG-CoA reductase inhibitor used for lipid lowering, also exhibits considerable anti-neoplastic activity. Although previous studies have shown that glucose starvation can potentiate several anticancer chemotherapies, atorvastatin has not been rigorously investigated for its impact on metabolic vulnerabilities and the effects on cholesterol-rich lipid rafts in aggressive tumors. This work aims to evaluate the combined anticancer activity of atorvastatin with metabolic interventions, specifically glucose starvation, on U-87 (glioblastoma) and MDA-MB-231 (triple-negative breast cancer) cell lines. **Methods:** U-87 and MDA-MB-231 cancer cells were cultured in either normal or glucose-free media and treated with different concentrations of atorvastatin. The impact of atorvastatin on these cancer cells was analyzed by examining cell viability, apoptosis, cell cycle, and changes in membrane order within lipid rafts. **Results:** This study found that glucose starvation increased the sensitivity of U-87 cells to atorvastatin by lowering IC_50_ values and eliciting arrest in the G1 phase of the cell cycle. MDA-MB-231 cells were less dependent on glucose for viability; however, atorvastatin consistently induced S-phase arrest across both metabolic states. Additionally, atorvastatin induced apoptosis in both U-87 and MDA-MB-231 cells, with the effect being more pronounced and dose-dependent in the fasting state with glucose. Interestingly, both Caspase-3 and Caspase-9 were consistently downregulated by atorvastatin in U-87 cells, regardless of the fasting state, corresponding to the induction of cell cycle arrest. Membrane lipid rafts exhibited decreased membrane order under glucose starvation, which was further decreased in response to atorvastatin in both cell lines, indicating a reduction in cholesterol. **Conclusions:** These results demonstrate that atorvastatin exhibits anticancer activity, characterized by both contextual and metabolic targeted effects, including a reduction in cancer proliferation, the triggering of cell cycle arrest via the downregulation of caspase pathways, and a decrease in membrane order. Notably, the combined activity of combining antilipemic agents with glucose-fasting provides potential metabolic strategies that could help create more effective and personalized approaches to cancer treatment.

## 1. Introduction

Cancer, one of the world’s major causes of death, has directed intensified research for decoding its core mechanisms, and controlling the metabolic reprogramming that makes a durable therapeutic response extremely challenging [1,2,3,4]. According to Douglas Hanahan and Robert Weinberg, the cancer hallmarks can be concluded into six main disturbances in cell physiology: autonomous growth signals, resistance to proliferation-inhibitory signals, evasion of programmed cell death (apoptosis), persistent angiogenesis, unlimited capacity for replication, tissue invasion, and metastasis [3,5]. The rapid and extensive proliferation of cancerous cells, along with the poor blood supply, leads to hypoxia and nutrient depletion. However, cancer cells adapted a metabolic reprogramming for energy production and biosynthesis to survive and continue tumorigenic processes under these challenging conditions [6].

Defective metabolism was first proposed as a main cause of cancer by Otto Warburg [6,7,8]. His theory elucidates that tumorigenesis initiates from insufficient respiration and views the abnormal aerobic production of lactate in tumor cells as an indicator of respiratory insufficiency [8]. In 1929, Herbert Crabtree confirmed and extended Warburg’s findings by highlighting the variation in glycolysis levels among different cancer types and also discovered variability in fermentation according to environmental or genetic factors [9]. Subsequent studies have intensively considered the oncogenic metabolic behavior and the role of cellular metabolism in tumorigenesis [10].

Cancer cells exhibit a high glucose demand due to the Warburg effect, which involves upregulating glycolysis and generating a high level of intermediates required for the biosynthesis of lipids, amino acids, and nucleic acids [11,12]. Although glutamine can serve as an alternative nutrient and ATP source, tumors frequently suffer shortages of both glucose and glutamine. In response, many cancer types exhibit metabolic plasticity, reducing their dependence on these pathways and maintaining growth through alternative energy sources [13,14,15,16]. Among these alternative pathways, the adaptive shift to lipid metabolism in several cancer types [17] is crucial for lipid membrane synthesis, energy production and storage, as well as cellular signaling [11,15].

In this context, several research studies have reported a strong correlation between elevated lipid synthesis and cancer progression, as well as the upregulation of various fatty acid uptake mechanisms [17,18,19,20]. Kuemmerle, N.B. et al. highlighted the expression of lipoprotein lipase (LPL) and the fatty acid uptake channel (CD36) in selected breast cancer and sarcoma cells, along with noticeable accelerated cell growth by the aid of LPL, in the presence of triglyceride-rich lipoproteins [21]. Furthermore, an upregulation of D-bifunctional protein (DBP) in the peroxisomal branched-chain fatty acid β-oxidation pathway has been reported in prostate cancer at both the mRNA and protein levels, accompanied by increased enzymatic activity [22]. This flexibility not only supports tumor survival under metabolic stress but also contributes to drug resistance and metastasis; thus, limiting lipid metabolism is a promising strategy for chemotherapeutic intervention [23,24].

Statins are extensively investigated lipid-lowering agents for their potential anticancer activity mediated by diverse mechanisms [25]. In particular, the lipophilic statins demonstrated a stronger pro-apoptotic activity [26] and better cellular uptake when compared to hydrophilic statins [25,26,27]. Atorvastatin is a lipophilic statin that has showed an evidenced efficacy in the treatment of several cancer types [28,29,30,31], owed to its activity in inducing autophagy [32,33] through activation of LC3 transcription [34] or independent of the MEV pathway [35], inhibiting pyroptosis [36], which is related to the development of cancer [25], apoptosis induction in hypoxia-induced cells with and without irradiation by reducing the HIF-1α protein expression [37] and promoting cell-cycle arrest of G1 phase by inhibiting Ras signaling pathways [38,39].

Several studies have revealed the role of atorvastatin in modulating lipid rafts, which contribute to its anti-inflammatory activity [40] and treatment-associated muscle symptoms [41]. However, its influence on lipid rafts in cancer cells is not well investigated. Since lipid rafts play a critical role in cancer cell proliferation, adhesion, and migration [42], the inhibition of these microdomains’ production by cholesterol depletion has antitumor effects [43]. This work aims to investigate the combined effect of glucose and lipid deprivation on the viability of cancer cells and the potential mechanism underlying atorvastatin’s anticancer activity.

## 2. Materials and Methods

### 2.1. Material

Cell lines, including U-87 (glioblastoma, ATCC: HTB-14-LUC2) and MDA-MB-231 (triple-negative breast cancer, ATCC: HTB-131), were obtained from the American Type Culture Collection (ATCC, Manassas, VA, USA). Minimum essential medium, penicillin, streptomycin, phosphate-buffer saline (PBS), L-Glutamine, and trypsin were obtained from EuroClone (Milan, Italy). Fetal bovine serum was obtained from Capricorn Scientific (Ebsdorfergrund, Germany), and DAPI dye from Thermofisher, Inc (Waltham, MA, USA). MTT reagent was obtained from Bioworld (Louis Park, MN, USA), and atorvastatin from Tabuk Pharmaceuticals (Tabuk, Saudi Arabia).

### 2.2. Cell Culture and Morphological Observations

Cell lines, including U-87 and MDA-MB-231, were cultured in minimal essential medium (MEM) under two different conditions: either supplemented with high glucose (25 mM, 4.5 g/L) or under glucose starvation (5.5 mM, 1 g/L). The media was supplemented with 10% FBS, 5% L-Glutamine, and 5% Penicillin/Streptomycin. Cells were passaged every 72 h and incubated at 37 °C, 5% CO_2_, and 90% relative humidity. Observations were taken considering the following parameters: (1) confluency in response to glucose starvation, (2) morphological changes, and (3) multinucleation. Microscopic images of cells were captured using an LCD light microscope screen at magnifications of 4×, 10×, and 20×.

### 2.3. Cell Viability

Cells were seeded in a 96-well plate at a density of 10 × 10^3^ cells/well and incubated at 37 °C, 5% CO_2_, and 90% humidity for 24 h. After that, cells were treated with a concentration range of atorvastatin (0.49–500 μM) and incubated for 24, 48, and 72 h, with eight replicates for each concentration at each time point. After incubation time, since atorvastatin was observed to cause cell detachment, two approaches were undertaken to assess viability of attached and detached cells, by either removing the media, keeping only attached cells, and treating each well with 90 μL media and 10 μL MTT reagent (3-(4,5-dimethyl-2-thia zolyl)-2,5-diphenyltetrazolium bromide), and incubating the cells for 3 h under the same conditions. After that, the media was removed and the formed crystals were solubilized with 50 μL of dimethyl sulfoxide (DMSO). Optical density was measured at a wavelength of 560 nm using a Glowmax microplate reader (Promega, Madison, WI, USA). The other approach, carried out to test whether detached cells are still viable, involved keeping the detached cells and treating them with 10 μL of MTT reagent. Followed by incubation for 3 h and using a stop solution prior to measuring the absorbance.

The feasibility of the assays at the various time points was ascertained during the first dose–response experiments for cell viability. From our observations, treatment with atorvastatin resulted in a significant number of cells falling off the plates entirely after 48 h. Thus, for accurate and repeatable data for the apoptosis, cell cycle, and RT-qPCR analyses, those experiments could only be performed at the 24- and 48 h time points, as the later time points would not have been reliable due to cellular loss. Untreated controls were consistently measured at all time points for each of the complete body of experiments, allowing for each to be used as a baseline comparison. The IC50 values for atorvastatin in both cell lines, under both glucose-fasting and non-fasting conditions, were determined from the data presented in Section 3.2. These calculated IC50 values were then used as the treatment concentrations for all subsequent experiments (apoptosis, cell cycle, and RT-qPCR).

### 2.4. Apoptosis

U-87 and MDA-MB-231 cells were seeded in 12-well plates under glucose fasting and non-fasting conditions at a seeding density of 3 × 10^5^ cells/well and incubated at 37 °C with 5% CO_2_ and 90% humidity for 24 h. After that, cells were treated with atorvastatin at IC_50_ and half-IC_50_ values in triplicate and incubated for 48 h under the same conditions. Cells were later collected and treated with FITC and PI staining buffers using TACSTM Annexin V detection kit (RnD Systems, Minneapolis, MN, USA) according to the manufacturer’s instructions. Apoptotic populations were measured using a FACS Canto II flow cytometer to detect emission peaks at 525 nm and 578 nm for FITC and PI, respectively. The data were analyzed using BD FACSDiva™ software version 8.0.

### 2.5. Cell Cycle

Cells were seeded in 6-well plates under fasting and non-fasting glucose conditions at a density of 5 × 10^5^ cells/well and incubated at 37 °C with 5% CO_2_ and 90% humidity for 24 h.

Cells were then treated with atorvastatin at IC50 values in triplicate and incubated for 48 h under the same conditions. After that, the cells were collected and centrifuged at 300× *g* for 5 min. The pellets were then washed with PBS, and fixation was carried out by gently adding the cell suspension to cold ethanol and incubating it for 15 min. Cells were centrifuged again, and the pellet was stained with 300 μL of propidium iodide (PI) staining buffer and incubated in the dark for 1 h. Finally, the fluorescence of PI was measured at an emission peak of 535 nm using a FACSCanto II flow cytometer (BD Biosciences, San Jose, CA, USA), and cell cycle analysis was performed using FCS Express 7 (Ruo edition).

### 2.6. RT-qPCR

Cells were seeded in 6-well plates under fasting and non-fasting glucose conditions at a seeding density of 5 × 10^5^ cells/well and incubated at 37 °C with 5% CO_2_ and 90% humidity for 24 h. After that, cells were treated with atorvastatin at IC_50_ values in triplicate and incubated for 48 h under the same conditions. Later, cells were lysed using the triazole-hybrid method and RNA was extracted using RNeasy Plus Mini Kit column (Qiagen, Germantown, MD, USA). The obtained RNA was quantified using a Nanodrop (Thermo Fisher Scientific, Waltham, MA, USA), and cDNA was synthesized using 1000 ng of total RNA with RT master mix (Takara, Kusatsu, Shiga, Japan) according to the manufacturer’s instructions. Reverse transcription was performed using a thermal cycler (Thermo Fisher Scientific, Waltham, MA, USA). RT-qPCR was carried out on CFX96 C1000 Touch thermal cycler (Bio-Rad, Hercules, CA, USA) by mixing 2 μL of the sample cDNA with 0.2 μL of forward primer, 0.2 μL reverse primer, 5 μL qPCR Master Mix (Promega, Madison, WI, USA), and 2.6 nuclease-free H_2_O. Temperature settings for the qPCR run were set at 95 °C for 3 min, followed by 40 cycles of 95 °C for 5 s and 62 °C for 30 s. For the melt curve, the temperature was set at 65 °C for 0.05 min, followed by 95 °C for 0.5 min. 18S rRNA was used as the reference gene, and data analysis was carried out in accordance with the 2^−ΔΔCq^ method.

### 2.7. Lipid Raft: Confocal Imaging and Analysis

Cells were seeded on sterile cover slips in 6-well plates under glucose fasting and non-fasting conditions at a seeding density of 5 × 10^5^ cells/well and incubated at 37 °C with 5% CO_2_ and 90% humidity for 24 h. After that, cells were treated with IC50-equivalent concentrations of atorvastatin and incubated for 24 and 48 h. After that, cellular plasma membranes were stained with 2:1000 Di-4-ANEPPDHQ in serum-free media and incubated for 1 h under the same conditions. Then, cells were fixed with 500 μL of 4% formaldehyde for 20 min at room temperature in the dark, followed by washing with PBS and treatment with 500 μL of ammonium chloride for 10 min in the dark at room temperature, followed by another wash with PBS. Cells were then stained with 500 μL of 1:1500 DAPI in PBS and incubated for 15 min in the dark at room temperature. The cells were finally washed with 500 μL PBS, and the cover slips were fixed, upturned on a glass slide, with one drop of mounting medium (Dako, Agilent Technologies, Santa Clara, CA, USA), and left to dry overnight. Stained samples were imaged using a Zeiss LSM780 confocal microscope system (Carl Zeiss AG, Oberkochen, Germany), with a 488 nm diode laser used to excite the Di-4-ANEPPDHQ stained, recording two fluorescent intensity channels: 500−580 and 620−750 nm. The mean fluorescence intensity was measured in the plasma membrane region of individual cells using ImageJ version 1.54 (NIH, Bethesda, MD, USA) with the GP (generalized polarization analysis) plugin (http://www.optinav.info/Generalized_Polarization_Analysis.htm (accessed on 2 June 2025)), modified to include a measured G (calibration factor) and to determine the general polarization (GP) value, assessing membrane order and disorder.

### 2.8. Statistical Analysis

GraphPad Prism 8 was used for thorough statistical analysis of all data. A two-tailed unpaired *t*-test was conducted to determine significance; significant differences were considered at a threshold of *p* ≤ 0.05. All data were collected in triplicate and presented as mean ± standard deviation (SD).

## 3. Results and Discussion

### 3.1. Morphological Observations

**Figure 1:**
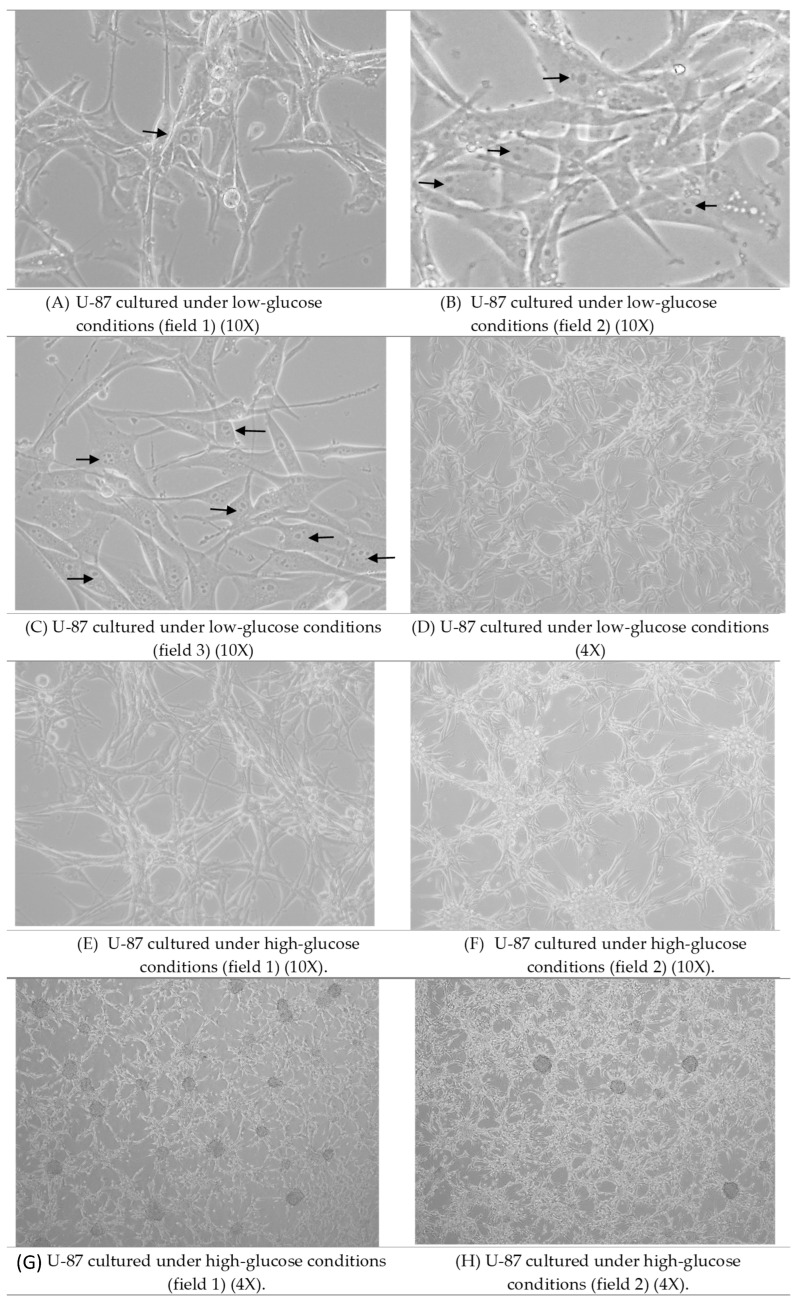
U-87 cell culture under (**A**–**C**) glucose-fasting conditions (20×), arrows indicating multinucleated cells, (**D**) glucose-fasting (10×), (**E**,**F**) glucose non-fasting conditions (20×), and (**G**,**H**) glucose non-fasting conditions (10×). Reduced confluency, multinucleation (arrows), and reduced axonal branching were observed in cells under low-glucose conditions.

Cell lines were cultured and observed under glucose-fasting and non-fasting conditions. U-87 cells exhibited key morphological alterations under glucose starvation (Figure 1A) compared with those cultured in high-glucose media (Figure 1B).

First, culture confluency is notably reduced compared to cultures under high-glucose conditions. This phenomenon has been observed every 72 h, indicating a glucose dependency in neural growth and survival. It has been previously established that glucose insufficiency reprograms neural metabolic pathways as neurons are most sensitive to and highly dependent on glucose availability, in which glycolysis is essential for synaptic activity and neural survival [44,45]. Furthermore, high glucose has been associated with increased aggressiveness in glioma, with increased cell growth and multiple alterations that facilitate aerobic glycolysis and glucose uptake [46,47,48]. Second, multinucleation is clearly observed in cells under conditions of glucose scarcity, a property that is infrequently seen under normal conditions. This phenomenon has been previously observed in cells under chemical or radiation-induced stress [49,50]. However, while some studies have reported several alterations in glioma cells in response to glucose starvation, the issue of multinucleation has not been previously addressed. Third, structural alterations are observed, with reduced axonal branching, whereas cultures under high glucose exhibit extensive branching. Higher glucose has been reported to increase axonal collateral branching, as glycolytic pathways play essential roles in axon extensions and growth [51,52].

**Figure 2:**
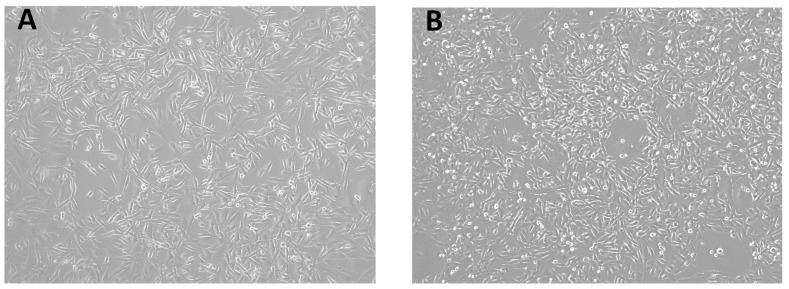
MDA-MB-231 cells cultured in (**A**) glucose-fasting conditions (4×) and (**B**) glucose non-fasting conditions (4×).

For MDA-MB-231 cells, no obvious morphological changes are observed upon culturing in glucose-fasting conditions (Figure 2). However, an evident increase in the rate of proliferation is identified, which was also reported by previous studies in which alterations in cellular growth and replication gene expression enhanced proliferation and facilitated malignancy [53,54]. This is supported by the S-phase measurements in Section 3.3, in which cells passaged under glucose-fasting conditions showed a total percentage in the S-phase population of 17.87%, while cells passaged under glucose non-fasting conditions showed a total percentage in the S-phase population of 21%. The increased population in S phase under glucose availability indicates the effects of metabolic stress on cellular proliferation, which is further elaborated in Section 3.3.

### 3.2. Cell Viability

The viability assay was conducted to investigate the effects of metabolic stress induction on U-87 and MDA-MB-231, focusing on glucose starvation and lipid starvation-like conditions induced by the antilipemic agent atorvastatin. To address the time dependency of atorvastatin exerting its cytotoxic effects, a viability test was conducted at three time points (24, 48, and 72 h).

**Figure 3:**
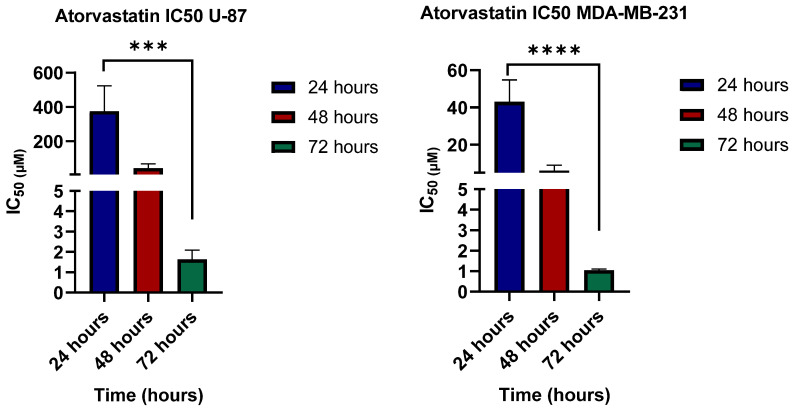
The cytotoxic effect of atorvastatin in U-87 and MDA-MB-231 cells under glucose-free conditions. Cell viability was assessed using the MTT assay after treatment with atorvastatin for 72 h. The data represents the viability of adherent cells only (*p* values = 0.0003 and <0.0001 for U-87 and MDA-MB-231, respectively). Statistical significance: *** *p* < 0.001; **** *p* < 0.0001.

Figure 3 shows the viability of U-87 and MBA-MB-231 in response to atorvastatin in a time-dependent manner. As indicated by the figure, a significant reduction in IC_50_ values is proportional to time. While different cell lines exhibit varying initial responses, a similar overall pattern was observed, as the cytotoxicity of atorvastatin increased with increasing exposure time to cells. While limited investigations have focused on these tumor cell-lines, it has been previously reported that while uptake of atorvastatin could take less than an hour [55], its anti-lipemic effects takes hours [56], and therefore, its antiproliferative effects takes ≥ 24 days [57], all of which is highly affected by the circumstances surrounding the cells and the heterogenicity of cancer cells, specifically.

**Figure 4:**
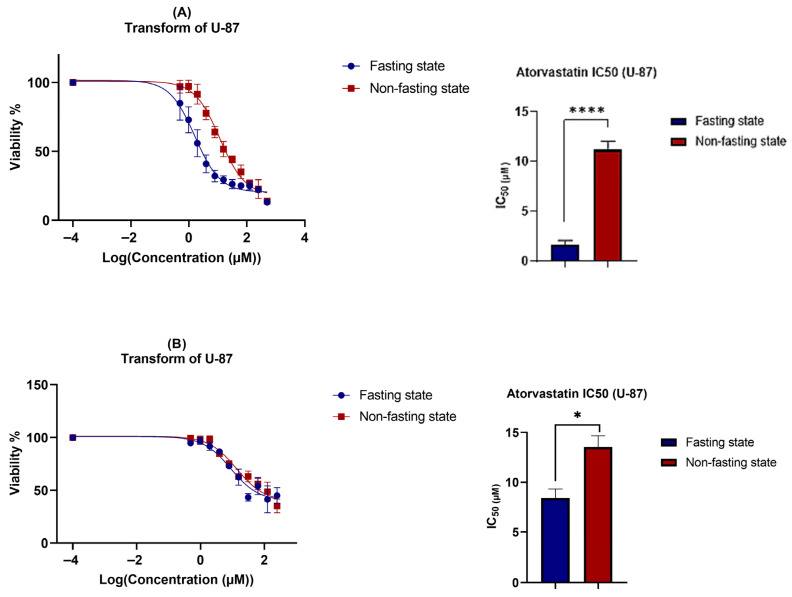
Combined effects of atorvastatin and glucose deprivation on U-87 cell viability. Cell viability was assessed using the MTT assay after treatment with atorvastatin for 72 h under both fasting (5.5 mM glucose) and non-fasting (25 mM glucose) conditions. The data in this figure were used to determine the IC50 values for each condition, which were then used as the treatment concentrations for all subsequent experiments (apoptosis, cell cycle, and RT-qPCR). (**A**) Viability of only the remaining attached cells. Atorvastatin IC50 values were 1.606 μM and 11.65 μM under fasting and non-fasting glucose conditions, respectively (*p* < 0.0001). (**B**) Viability of all cells (attached + detached). Atorvastatin IC50 values were 8.92 μM and 13.45 μM under glucose-fasting and non-fasting conditions, respectively (*p* = 0.0125). Statistical significance: * *p* < 0.05; **** *p* < 0.0001.

The cytotoxicity of atorvastatin was assessed in a concentration-dependent manner after 72 h of exposure to atorvastatin under two conditions: glucose fasting and non-fasting states. For accurate consideration, it has been observed that atorvastatin strongly induces cellular detachment, raising the question of whether the detached cells remain viable, undergo apoptosis, or remain arrested. To address this question, the viability test was repeated under two conditions: either by removing detached cells after the incubation period and measuring the viability of the remaining attached cells, or by keeping the suspended cells and measuring the viability of all cells (both detached and attached). Figure 4A shows U-87 viability and Atorvastatin IC_50_ without keeping the suspended, detached cells, and Figure 4B shows the same parameters keeping the suspended cells.

A notable decline in Atorvastatin IC_50_ values (1.606 μM) is observed under glucose scarcity, with an almost 7-fold reduction compared to its IC_50_ under non-fasting glucose conditions (11.65 μM). These results indicate that glioma vulnerability to atorvastatin can be sensitized by glucose starvation. While no previous data are available regarding the effects of this antilipemic agent on glioma under glucose starvation, it has been reported that U-87 cells cultured under similar conditions exhibit increased vulnerability and enhanced susceptibility to treatment [58]. Additionally, treatment resistance and tumor aggressiveness are often associated with increased glycolysis [59]. It has also been reported that atorvastatin exerts its effects on glioma in a concentration-dependent manner, exerting its antiproliferative effects at concentrations ranging from 5 to 20 μM under normal metabolic conditions [60,61]. Another important observation is the increase in IC50 values when keeping the detached cells, with an overall reduction in viability of 60% compared to a total reduction of almost 80% in the first method. This could be explained, at least in part, by a potential cycle arrest pattern, which is further elaborated on in the cell cycle Section 3.3, where cells exhibited cycle arrest in response to Atorvastatin treatment.

**Figure 5:**
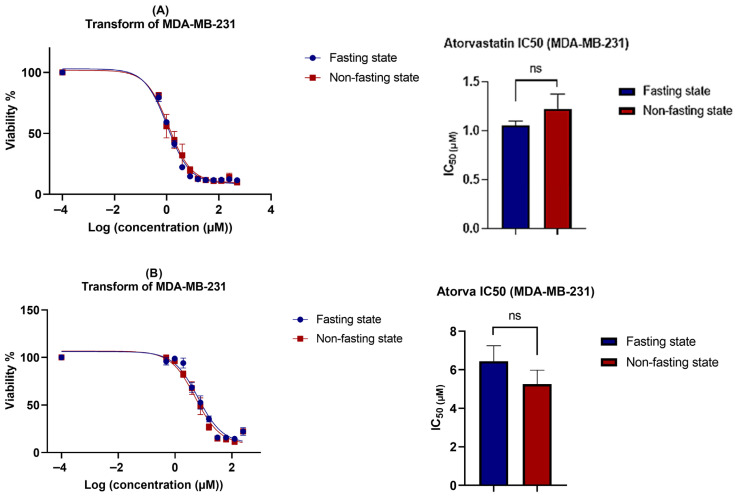
Viability of MDA-MB-231 cells after 72 h in response to atorvastatin. Cell viability was assessed using the MTT assay after treatment with atorvastatin for 72 h under both fasting (5.5 mM glucose) and non-fasting (25 mM glucose) conditions. (**A**) Viability of only remaining attached cells. Atorvastatin IC50 values were 1.052 μM and 1.186 μM under fasting and non-fasting glucose conditions, respectively (*p* = 0.343). (**B**) Viability of all cells (attached + detached). Atorvastatin IC50 values were 6.44 μM and 5.258 μM under glucose-fasting and non-fasting conditions, respectively (*p* = 0.3142). Statistical significance: ns = not significant.

Similarly, MDA-MB-231 showed high susceptibility to Atorvastatin with IC_50_ values ~1 μM (Figure 5). These results are consistent with previous reports on Atorvastatin IC_50_ in breast cancer cell lines, ranging from 2 to 50 μM, with time-dependency and a reduction in IC_50_ ≤ 5 μM after 72 h of exposure [57,62]. However, little glucose dependency was observed, as non-significant differences were obtained upon treatments under glucose-fasting and non-fasting conditions. A similar pattern was observed when measuring the IC_50_ values by keeping the suspended cells or removing them, which is further explained by the cell cycle analysis that follows.

### 3.3. Cell Cycle

**Figure 6:**
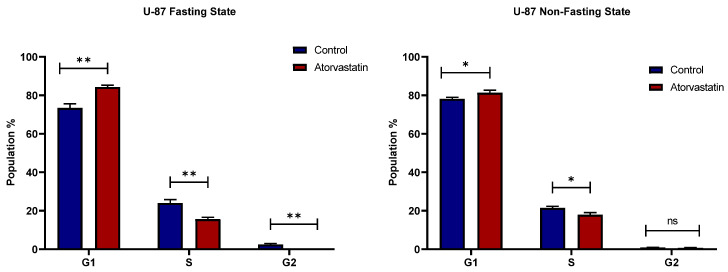
U-87 cells also undergo a G1 phase arrest following treatment with atorvastatin, and glucose deprivation further enhanced this arrest when combined with atorvastatin. The cell cycle profile was analyzed following 48 h of treatment with atorvastatin in glucose-fasting and glucose-fed states. We could observe a meaningfully significant shift in the cell population. The quantification of the cell cycle phases indicated a significantly increased G1 population and reduced S phase population relative to controls, especially in the combined treatment. The data is indicated as mean ± SEM of three independent experiments, and the statistical analysis were performed by comparing treated cells to the corresponding untreated control cells. Statistical significance: ns = not significant; * *p* < 0.05; ** *p* < 0.01.

To determine the effects of atorvastatin on cell cycle progression, U-87 cells were treated with the IC50 concentration of atorvastatin for 48 h, as determined from our viability assays, and untreated cells served as controls. Cell cycle analysis suggested that atorvastatin induced cell cycle repression, resulting in a more significant shift towards early-stage arrest under glucose-starved conditions. Figure 6 depicts the cell cycle distribution of U-87 cells after 48 h of treatment with atorvastatin IC50, both under glucose-fasting and non-fasting states, alongside untreated controls.

**Figure 7:**
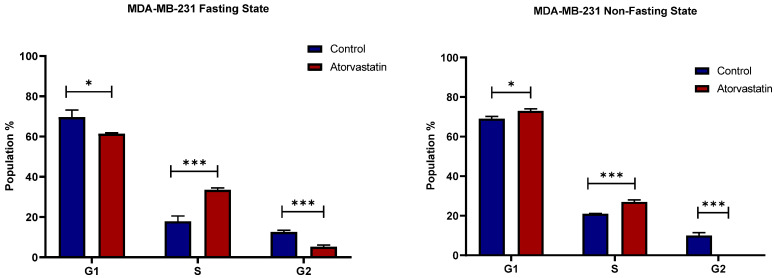
Cell cycle of MDA-MB-231 in response to atorvastatin under glucose fasting and non-fasting conditions. A shift in cell cycle arrest is observed towards the G1 phase under glucose starvation. Statistical significance: * *p* < 0.05; *** *p* < 0.001.

The cell cycle distributions revealed a variety of noteworthy visual changes, and while minimal visual differences can often represent statistically significant and biologically relevant changes for these two cell lines, our analysis showed significant changes in both cell lines. In U-87 cells treated with atorvastatin for 48 h we observed a statistically significant rise in the G1-phase population (e.g., compared to the untreated control *p* < 0.05) and a concurrent decline in the S and G2/M populations Figure 6. This G1 arrest appears most robust under glucose-fasting conditions, where the combined treatment with atorvastatin appears to be maximal, thereby associating this cellular mechanism with the reduction in cell viability observed. Alternatively, atorvastatin significantly affected the cell cycle in MDA-MB-231 cells with a consistent S-phase arrest independent of glucose conditions (e.g., compared to the untreated control, *p* < 0.01) Figure 7. The distinct cell cycle effects for each line provide important mechanistic insights into the differences in atorvastatin effects and connect directly with our viability and apoptosis data.

In the fasting State (left panel), treatment with atorvastatin modified the cell cycle profile, with a profound and significant increase in G1 phase population (from ~74% in control samples to 84% in Atorvastatin-treated samples; *p*: 0.0013) and a concomitant and considerable reduction in S phase cells (from ~24% to 16%; *p*: 0.0019). Additionally, an impact on G2-phase population is observed, with a reduction from (~2.5% to <0.5%; *p*: 0.001). Collectively, these changes suggest that Atorvastatin induces G1 cell cycle arrest in U-87 cells under glucose-fasting conditions.

In contrast, at the glucose non-fasting state, cycle arrest of U-87 appears at a later G1/S phase, but again, the effects were less pronounced than in the fasting state. Atorvastatin was still statistically significantly increasing the G1 phase population (comparing the control of ~approximately 79% to the Atorvastatin-treated group of 81%; *p* = 0.024) and resulting in a significantly different decrease in the S phase population (from 21% to 18%; *p* = 0.0113). However, the change for G1 and S phases is less significant than the change observed in cells at the glucose fasting state. The data also indicate no significant difference at the G2 phase between the control and Atorvastatin-treated groups (*p* = 0.3615). Overall, comparing the effects of Atorvastatin on U-87 cells under glucose-fasting and non-fasting states, a cycle arrest is observed, with an obvious shift to an earlier arrest prior to the S and G2 phases under glucose starvation, indicating an enhanced effect when atorvastatin was combined with glucose starvation on U-87 cells. While atorvastatin has been reported to induce cell cycle arrest in glioma [63], these results highlight its enhanced effects when combined with the metabolic state of the cells.

The Impact of atorvastatin on the cell cycle distribution of MDA-MB-231 cells is shown in Figure 7 for both fasting (left panel) and non-fasting (right panel) states. Under glucose starvation, treatment with atorvastatin significantly alters the cell cycle profile compared to the untreated control. A statistically significant decrease in the percentage of cells in the G1 phase (control ~70% compared to Atorvastatin-treated 60%; *p* = 0.0166). Alternatively, a considerable and statistically significant increase in S phase population (control ~17% vs. Atorvastatin 35%; *p* = 0.0007). The percentage of G2 cells was also significantly decreased (from ~approximately 15% to 5%; *p* = 0.0006). These observations suggest that cycle arrest occurs at the S phase in MDA-MB-231 cells, rather than an earlier arrest at the G1 phase, with potential retraction from the G2/M phase. A similar pattern is observed in the non-fasting state of glucose. There is again a statistically significant decrease in the G1 phase population with atorvastatin (from ~69% in control to 72% in Atorvastatin-treated group; *p* = 0.0116), and a statistically significant increase in the S phase population (from ~21% to 27%; *p* = 0.0006). Similarly, a reduction in the G2 phase population from (~9% to < 0.5%; *p*: 0.0002) is observed. Similar effects were reported of cycle arrest in response to atorvastatin at the S phase in TNBC, without specification on the metabolic state of the cells [64].

The effects of atorvastatin on cell cycle progression in U-87 and MDA-MB-231 cell lines under both fasting and non-fasting glucose states demonstrated opposite effects in each cell line, with subtle differences in drug sensitivity and outcomes, particularly under contrasting metabolic conditions. In U-87 cells, atorvastatin induced a cycle arrest at the G1 phase, as indicated by an increase in the G1 population and a decrease in the S and G2 populations. The effect was greater in a fasting state, suggesting that atorvastatin can inhibit cell proliferation in this glioblastoma cell line with an enhanced effect under fasting conditions. This finding was consistent with prior studies that suggested metabolic stress can enhance the effectiveness of certain agents by altering pathways related to growth and survival [65,66]. Specifically, caloric restriction has been reported to enhance the antitumor therapeutic benefit of various therapies by altering the metabolic reprogramming and stress responses of cancer cells [67].

In contrast, MDA-MB-231 exhibited a distinct form of cell cycle perturbation following treatment with atorvastatin, characterized by cycle arrest at the S phase in both metabolic conditions. This indicates that cells are either mobilizing additional resources for cellular division in S phase, or they are in S-phase arrest or delay. Thus, even with overall trends of S-phase accumulation in both fasted and non-fasted states, the magnitude of the decrease in G1 and the accumulation in S phase appeared to be somewhat greater in the fasted state than in the non-fasted state, but still not pronounced as the G1 arrest induced by atorvastatin in U-87 cells.

The observation of cellular cycle arrest at different cell cycle points, in response to the modulatory influence of fasting glucose, underscores the need to consider the complexity of the cellular context and the impact of the metabolic environment when evaluating any therapeutic action. Furthermore, the observed differences in cycle-arrest phases imply that the action of atorvastatin and its relationship to fasting may be tumor-type specific, laying the groundwork for further analysis of changes in cellular proliferation in the cell cycle in the future. These findings could help pave the way for individually tailored therapeutic approaches that combine nutritional and metabolic interventions with pharmacological interventions to target their specific pro- and anticancer effects across different malignancies.

### 3.4. Apoptosis

**Figure 8:**
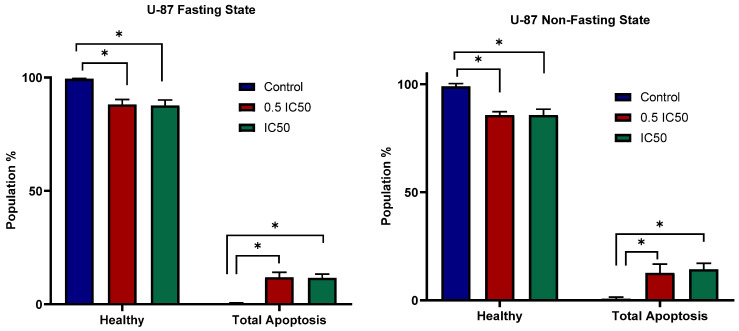
Apoptotic effects of Atorvastatin on U-87 cells under glucose fasting and non-fasting conditions. Statistical significance: * *p* < 0.05.

To evaluate the effect of atorvastatin on apoptosis, cells were treated with the IC50 of atorvastatin for 48 h, as determined by our viability studies, with corresponding untreated controls included at this time point. The apoptotic effects of Atorvastatin on U-87 and MDA-MB-231 were studied at two concentrations (0.5 IC50 and IC50 values). Figure 8 shows U-87 populations at the glucose non-fasting (left panel) and glucose-fasting (right panel) effects. In both cases, control cells show a healthy population ≥99%. However, induction of apoptosis was observed in response to atorvastatin with similar effects at both concentrations. In comparison with controls in the non-fasting state, a total healthy population of ~87% and an apoptotic population of ~13% (* *p* = 0.0108) are observed in response to 0.5 IC50 values. Of the total apoptotic population, early apoptosis accounted for 4.7%, late apoptosis for 6.75%, and necrosis for 2.9%. A similar pattern is observed in response to IC50 values, with a total healthy population of ~86% and a total apoptotic population of ~14% (*p* = 0.0111). Of the total apoptotic population, early apoptosis comprised 4.4%, late apoptosis 7.0%, and necrosis 2.9%. With no obvious differences observed across the two studied concentrations (*p* = 0.8772), a plateau in the apoptotic effects is reached in the dose–response curve.

Similarly, in the U-87 Fasting State (right panel), the control maintains almost 99% healthy cells. Treatment with 0.5 IC50 reduces the healthy cell population to approximately 88% and results in an increase in total apoptosis of about 12% (*p* = 0.0355) compared to the control. Of the total apoptotic population, early apoptosis accounted for 4.8%, late apoptosis for 4.6%, and necrosis for 2.5%. The IC50 treatment reduces healthy cells to approximately 88% and increases total apoptotic levels to about 12% (*p* = 0.0156) compared to the control. Of the total apoptotic population, early apoptosis accounted for 4.7%, late apoptosis for 6.5%, and necrosis for 1.2%. Once again, similar to the non-fasting state, treatment with both the 0.5 IC50 and IC50 concentrations did not result in a statistically significant difference in the healthy cell population or the amount of total apoptosis (*p* > 0.9999).

**Figure 9:**
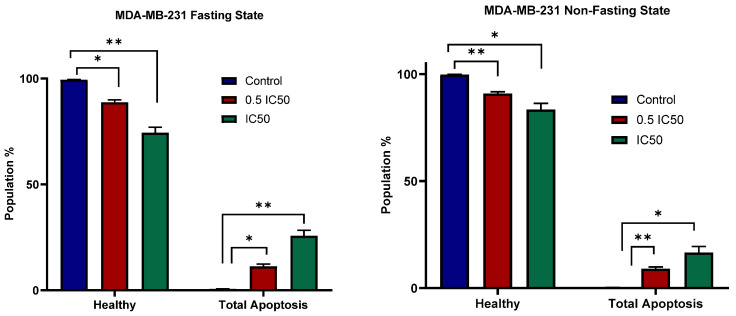
Apoptotic effects of Atorvastatin on MDA-MB-231 cells under glucose fasting and non-fasting conditions. Statistical significance: * *p* < 0.05; ** *p* < 0.01.

Figure 9 shows the percentage of healthy and total apoptotic MDA-MB-231 cells exposed to 0.5 IC50 and IC50 concentrations of atorvastatin under non-fasting and fasting conditions. In the case of the MDA-MB-231 Fasting State (left panel), the control group had almost 99% of the cells remaining healthy with minimal total apoptosis. At a 0.5 IC50 dosage, the population of healthy cells decreased to approximately 89%, and total apoptosis was significantly induced to 11% (*p* = 0.0104) compared to the control. Of the total apoptotic population, early apoptosis accounted for 4.5%, late apoptosis for 5.7%, and necrosis for 1.1%. At the higher IC50 concentration, there was a significant decrease in the number of remaining healthy cells in the unfasted state to approximately 74%, and a significant increase in total apoptosis to around 26% (*p* = 0.0041 compared to the control). Of the total apoptotic population, early apoptosis accounted for 8.5%, late apoptosis for 15%, and necrosis for 2.2%. There was a clear dose-dependent increase in apoptosis. We can see that MDA-MB-231 cells are also dependent on fasting-state conditions to improve therapeutic outcomes.

In the non-fasting state of MDA-MB-231 (right panel), the control has nearly 99% of healthy cells and a very small percentage of total apoptosis. The treatment with 0.5 IC50 reduced the healthy cell portion to approximately 91% and increased the total apoptosis to approximately 9% (*p* = 0.0031) compared to the control. Of the total apoptotic population, early apoptosis accounted for 2.4%, late apoptosis for 5.9%, and necrosis for 0.8%. When the concentration was increased to the IC50, the healthy cell portion decreased appreciably to approximately 83%, and the total apoptosis increased significantly to approximately 17% (*p* = 0.0102 compared to the control). Of the total apoptotic population, early apoptosis accounted for 4.6%, late apoptosis for 10.8%, and necrosis for 1.1%. These results indicate a dose-dependent total apoptosis in the non-fasting state in MDA cells.

When comparing both conditions, atorvastatin induces apoptosis in MDA-MB-231 cells in both the non-fasting and fasting conditions; however, the apoptotic effect appears to be more pronounced, with a steeper dose–response, in the fasting state. At IC50, total apoptosis in the fasting state is higher (approximately 26%) compared to the non-fasting state (approximately 17%), indicating that fasting may sensitize MDA-MB-231 cells to apoptosis. This stronger apoptotic effect in the fasting state for MDA-MB-231 cells contrasts with the U-87 cell line, where the fasting condition did not significantly alter atorvastatin’s ability to induce apoptosis.

The study of atorvastatin’s apoptotic effects on U-87 and MDA-MB-231 cell lines reveals significant differences in their characteristics in response to fasting conditions. Atorvastatin had an apoptotic effect on U-87 cells in both non-fasting and fasting conditions; in each case, atorvastatin reduced the number of healthy cells and increased the total amount of apoptosis at both the 0.5 IC50 and IC50 concentrations. It is important to note that the total amount of apoptosis had a maximum response, and the difference between concentrations was not significantly different; fasting conditions did not substantially modify atorvastatin’s ability to induce cell death in this glioblastoma cell line. This finding suggests that although U-87 cells appear to be susceptible to an apoptosis effect from atorvastatin, the degree to which this occurs is largely invariant to nutrient conditions during the tested concentrations. The importance of this finding lies in the fact that glioblastomas are highly malignant cancers and are frequently not responsive to therapies; thus, identifying treatment factors that consistently induce apoptosis is crucial [68].

By contrast, the TNBC cell line, MDA-MB-231, exhibited a dose-dependent increase in atorvastatin-induced apoptosis in both non-fasting states and fasting states. Notably, even though the MDA-MB-231 cells demonstrated some apoptosis in the non-fasting state, the response was significantly enhanced in fasting states, and the dose–response curve was steeper. This was identified at the IC50 concentration; fasting states resulted in a larger percentage of total apoptosis compared to non-fasting states. Thus, fasting has been shown to sensitize MDA-MB-231 cells towards atorvastatin-induced apoptosis. This unique finding, which differentiated the cell lines, is that fasting sensitized the MDA-MB-231 cells but not the U-87 cells.

It emphasizes that the relationship between metabolic paradigms (glucose-fasting) and pharmaceuticals, including atorvastatin is highly contextual and cell-line specific. This cell-line-specific context suggests that there may be a need for more personalized approaches that incorporate metabolic modulation, along with targeted drug therapy, in optimal therapeutic strategies for various types of cancer. The results are also in line with increasing evidence that there are metabolic vulnerabilities one can exploit for cancer patients and that dietary approaches can enhance the effects of anticancer drugs in cancer models, especially as it relates to breast cancer [69,70]. Overall, this work may contribute to the understanding of how drug efficacy changes in different metabolic states and lays the groundwork for more tailored cancer treatments, laying the grounds for further future work focusing on apoptotic confirmation in response to atorvastatin-mediated metabolic stress.

### 3.5. Gene Expression

In our viability studies, the IC50 of atorvastatin was determined in U-87 and MDA-MB-231 cells. To assess gene expression, we treated both cell lines with the atorvastatin IC50 dose for 48 h. Untreated controls were included at the 48 h time point. The role of atorvastatin was assessed by evaluating gene expression of key apoptotic regulators and cell cycle regulators in the U-87 and MDA-MB-231 cells. We found that atorvastatin treatment at its IC50 dose resulted in a significant downregulation of Caspase-3 and Caspase-9 genes in both U-87 and MDA-MB-231 cells, regardless of the cell line, indicating a potential effect of downregulating these genes, which may depend on the fasting and non-fasting metabolic status. We also assessed gene expression in several other important regulators, such as the anti-apoptotic protein BCL-2, the cell cycle inhibitor p21, and the proto-oncogene c-MYC. Although these genes were assessed for gene expression in the U-87 and MDA-MB-231 cell lines, no expression was observed in any of the controls or treatment samples.

**Figure 10:**
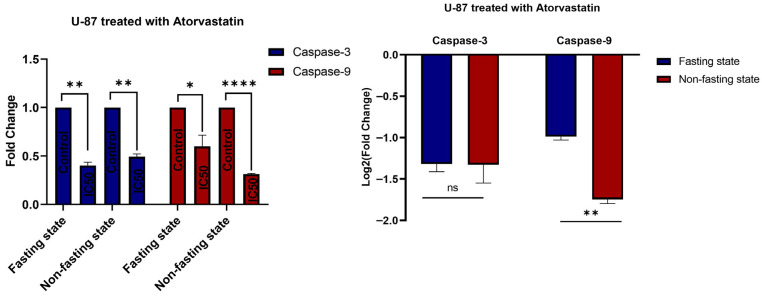
Expression of Caspase-3 and Caspase-9 in response to atorvastatin at glucose-fasting and non-fasting states in U-87 cells. Statistical significance: ns = not significant; * *p* < 0.05; ** *p* < 0.01; **** *p* < 0.0001.

In U-87, Atorvastatin treatment resulted in a statistically significant reduction in Caspase-3 gene expression compared to controls, both in fasting and non-fasting states (Figure 10). Specifically, in fasting conditions, Caspase-3 expression decreased by approximately 0.4-fold (*p* = 0.0018) relative to the control. In the non-fasting condition, Atorvastatin treatment resulted in a 0.5-fold reduction in caspase-3 expression (*p* = 0.0014) relative to the controls. This clear, consistent, and statistically significant down-regulation of Caspase-3, an executioner caspase in the apoptotic cascade, potentially indicates a direct impact on the process of programmed cell death in U-87 cells, regardless of the immediate metabolic environment. Atorvastatin also displayed notable downregulation of Caspase-9 in U-87, an initiator caspase involved primarily in the intrinsic (mitochondrial) apoptotic pathway, yielding promising values for modulation. In the fasting condition, it had decreased levels of Caspase-9 expression to ~0.6-fold (*p* = 0.039) of the control. It was even more alarming that, in the non-fasting state, atorvastatin treatment resulted in a pronounced decrease in caspase-9 expression, to ~0.3-fold (*p* < 0.0001) of the control. The pronounced suppression of Caspase-9 observed in the non-fasting state suggests that atorvastatin may suppress the initiation of the intrinsic apoptotic pathway more when nutrients are readily available or cells are actively metabolizing, rather than in the fasting state.

The observed downregulation of Caspase-3 and Caspase-9 in U-87 glioblastoma cells, attributed to atorvastatin, is noteworthy. This is especially noteworthy, given that caspases are hallmarks of apoptosis, and cancer therapies often activate them. However, the area of statins and cancer is complicated and diverse. While studies report pro-apoptotic or antiproliferative effects, there are also studies documenting the possible inadvertent promotion of survival or varied cellular activities in various cancers or specific conditions [71,72]. The preferential downregulation of Caspase-3 and Caspase-9 expression suggests a nuanced effect of atorvastatin.

The current results suggest that atorvastatin enables U-87 cells to act in a manner that potentially inhibits the direct induction of apoptosis through the canonical caspase pathways at the gene level. Atorvastatin acts primarily as an HMG-CoA reductase inhibitor, which affects decreases cholesterol formation and the products of the mevalonate pathway. These products are essential for the prenylation of proteins, which include small GTPase regulators such as Ras and Rho that regulate cell growth, survival, and proliferation [73,74,75]. Conversely, disrupting these pathways may affect the expression of apoptotic machinery genes in a unique way, and cancer may demonstrate adaptation or compensatory pathways.

Fasting or a lack of nutrients can alter cellular metabolism and stress responses, potentially increasing cell susceptibility to drugs [76,77]. The study did show a greater inhibition of Caspase-9 in the non-fasting state, which could imply that, given active metabolism and perhaps a greater dependence on products from the mevalonate pathway for rapid growth, atorvastatin has a stronger effect on the related cellular survival pathways involving Caspase-9.

Atorvastatin’s complex response in U-87 glioblastoma cells included strong G1 cell cycle arrest, substantial apoptosis, and simultaneous downregulation of several caspase gene expressions, all of which present an intriguing biological puzzle for us to ponder. Atorvastatin consistently induced a strong G1 phase cell cycle arrest in U-87 cells, with an even stronger effect observed under fasting conditions. This cell cycle arrest would, therefore, lead to an inhibiting mechanism that affects cellular proliferation; in other words, it is a well-known strategy for cancer therapy that prevents cells from dividing and subsequently stops the replication of their DNA [78]. Given such a strong antiproliferative effect, one would imagine that apoptotic pathways would be rather simple to activate in these arrested cells to remove or eliminate them.

Certainly, atorvastatin induces a significant level of apoptosis in U-87 cells, which is a clinically important outcome related to anticancer efficacy. However, the downregulation of Caspase-3 (executioner) and Caspase-9 (initiator) gene expression by atorvastatin is particularly surprising. Traditionally, we consider caspases to be the key mediators of apoptosis, meaning that caspases are considered to initiate apoptosis by increased expression and cleavage [79,80]. While some studies suggest that caspases, through the cleavage of cell cycle regulatory proteins, can impact cell cycle progression, their primary function is as a cell death protease. Some studies suggest that, in particular, caspase-3 and caspase-9 may play a role in regulating the transition from mitosis to the G1 phase, but this is not their primary role [81]. The fact that apoptosis can be induced while the transcriptional expression of caspase genes is downregulated suggests a more complicated and potentially caspase-independent mechanism or simply a mechanism where the caspase is activated post-transcriptionally, thereby disregarding reduced diploid expression. Secondly, perhaps one consequence of a prolonged G1 arrest, possibly under the metabolic stress of fasting, will drive the cells towards alternate forms of programmed cell death, such as necroptosis or autophagy-dependent cell death that do not directly depend on the transcriptional upregulation of specific caspases in normal cellular metabolism [82,83].

As a result, the relationship between G1 arrest, caspase downregulation, and apoptosis in U-87 cells is not a simple linear cascade. It suggests that atorvastatin may be driving cells into a non-replicative state via G1 arrest and killing cells via cellular pathways that may not require increased gene expression of apoptosis-associated caspases or involve post-transcriptional regulation that is probably acting in a sophisticated manner. The interaction between fasting and G1 arrest was more pronounced, but not solely by increasing the level of apoptosis or driving caspase gene expression in a linear, pro-apoptotic manner, further affirming the complexity and context-dependence of atorvastatin activity in glioblastoma cells and the separately interesting combination with fasting. Indeed, the interesting complexity is peculiar and points to the novelty of these results, as well as the need to further characterize the underlying molecular events that define atorvastatin’s anticancer effects in U-87 cells, particularly in terms of the extent of caspase-dependent versus caspase-independent cell death, and the metabolic state of cells during treatment.

**Figure 11:**
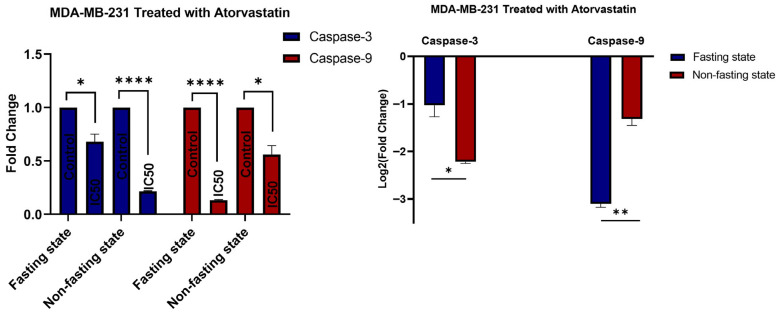
Expression of Caspase-3 and Caspase-9 in response to atorvastatin at glucose-fasting and non-fasting states in MDA-MB-231 cells. Statistical significance: * *p* < 0.05; ** *p* < 0.01; **** *p* < 0.0001.

In MDA-MB-231 cells (Figure 11), atorvastatin also downregulated the gene expression of caspase-3, albeit with a stronger effect on the gene expression of caspase-3 in the non-fasting state (approximately 0.2-fold, *p* < 0.0001) compared to the fasting state (approximately 0.25-fold, *p* = 0.0236). The unexpectedly larger downregulation in Caspase-3 in the MDA-MB-231 cells in the non-fasting state was an observed difference from U-87 cells, where the effect on Caspase-3 expression was less variable between states. Caspase-9 expression in MDA-MB-231 cells was significantly downregulated in both states, approximately 0.25-fold (*p* < 0.0001) in the fasting state and by approximately 0.1-fold (*p* = 0.0172) in the non-fasting state. The extreme downregulation of Caspase-9 in non-fasted MDA-MB-231 cells appears to be the single largest effect among both caspases and both cell lines studied, providing a strong case that this pathway and state may be a particularly vulnerable point of action for atorvastatin in this aggressive breast cancer subtype.

In our investigation of MDA-MB-231, we observed a unique and complex reaction to atorvastatin treatment, which differed from that of U-87 cells, particularly in terms of how the cell cycle, apoptosis, and caspase gene expression were affected under various metabolic conditions.

When examining the cell cycle, atorvastatin induces S-phase arrest or a delay in MDA-MB-231 cells. We know this from the statistically significant drop in G1 population, a large and significant increase in the S phase population, and the resultant decrease in G2 cells. These results were achieved in both fasting and non-fasting conditions. Although the direction of these changes is the same, there exist subtle differences in results between fasting and non-fasting metabolic conditions. The S-phase accumulation suggests that atorvastatin inhibited DNA synthesis or progression through the S-phase, a common antiproliferative mechanism reported in the literature [84,85,86].

In addition to the cell cycle data, the apoptosis assay reveals that atorvastatin exhibits a dose-dependent apoptosis-inducing effect on MDA-MB-231 cells, regardless of whether the cells are fasting or non-fasting. However, the apoptotic effect was more potent and had a steeper dose–response curve in the fasting state. At the IC50 concentration, total apoptosis was approximately 24% in the fasting state compared to approximately 16% in the non-fasting state. The increase in sensitivity to apoptosis in the fasting state suggests that starving MDA-MB-231 cells of nutrients, in conjunction with atorvastatin treatment, creates opportunities for metabolic vulnerabilities that trigger cell death [65,87,88].

Nonetheless, the gene expression study surrounding caspases presents a notable paradox. Noteworthy apoptosis occurred after the treatment with atorvastatin; however, there was a significant downregulation of both Caspase-3 and Caspase-9 gene expression in the MDA-MB-231 cells in both metabolic states. The downregulation of Caspase-3 expression was significantly lower in the non-fasted metabolic state (about 0.2-fold) compared to the fasted metabolic state (about 0.25-fold), while Caspase-9, the important initiator for the mitochondrial apoptotic pathway, was significantly downregulated to about 0.25-fold in the fasted state and only 0.1-fold in the non-fasted state (the most extreme finding among the studies). While caspases have been strongly downregulated at the transcriptional level, a clear S-phase arrest and high levels of apoptosis were still observed in the atorvastatin-treated MDA-MB-231 cells; therefore, the mechanism of induced cell death by atorvastatin in the MDA-MB-231 cell may be consistent with the proposed alternate mechanisms of cell death (caspase-independent mechanisms), or it could support the notion of post-transcriptional activation of existing caspase proteins [89,90,91,92].

This drastic transcriptional suppression of caspases (and caspase genes)—which occurs in parallel to S-phase arrest and extensive apoptosis—suggests that the mechanism of cell death in MDA-MB-231 cells by atorvastatin may occur via caspase-independent mechanisms or that the existing caspase proteins within the cells are activated post-transcriptionally [89,93,94]. Given the strong apoptotic effect observed during fasting, in conjunction with the transcriptionally extensive downregulation of caspase genes, this raises the possibility that fasting may promote cell death via mechanisms that are wholly independent of transcriptionally mediated induction of these classical caspases, which necessitates further investigation. Significant to the novelty of this investigation is the finding that an anticancer agent can lead to significant cell death and cell cycle arrest while simultaneously downregulating the gene expression of the key apoptotic caspases, especially in this context (for example, fasting promotes enhanced apoptosis despite downregulation of caspases). Therefore, there is an important link between the distinct mechanisms that must be considered, and further work is warranted to characterize the mechanisms by which atorvastatin acts in aggressive breast cancer, as well as its potential to promote co-treatments in conjunction with metabolic interventions.

### 3.6. Lipid Raft

The consideration of whether lipid rafts could be useful for modeling the cancer cell membrane, particularly while subjected to glucose deprivation and Atorvastatin treatment, is logical. Lipid rafts serve as essential and dynamic signaling platforms for both ordered and disordered domains. Lipid rafts are highly ordered membrane microdomains rich in cholesterol and sphingolipids that float within the more fluid, disordered bulk membrane. Thus, this inherent heterogeneity present in membrane topology provides orientation and compartmentalization of proteins involved in key cellular processes [95,96].

A lack of glucose profoundly alters cellular metabolism, which, in and of itself, can lead to alterations in lipid biosynthesis and composition that could influence the order and disorder of membrane structure and dynamics, thereby affecting the ordering and influx/lysis of lipid rafts. Atorvastatin affects cholesterol biosynthesis, which plays a direct role in the integrity and stability of ordered raft domains and may possibly promote a less ordered state in the surrounding membrane [97,98,99]. This work in examining lipid rafts under similar metabolic conditions allows us to explore how the combined effects of metabolic stress and lipid-lowering drugs, such as atorvastatin, interact with and impact the delicate ordering of membrane lipids in complex with ordered rafts, and consequently determine raft-related membrane protein localization and signaling pathways responsible for cancer cell survival, drug resistance, and metabolic adaptation. Although it is important to note that there has been conflicting data regarding the role of lipid rafts in promoting the localization of membrane protein signaling and the interplay of modulation of this mechanism by changes in lipid components [100,101,102].

**Figure 12:**
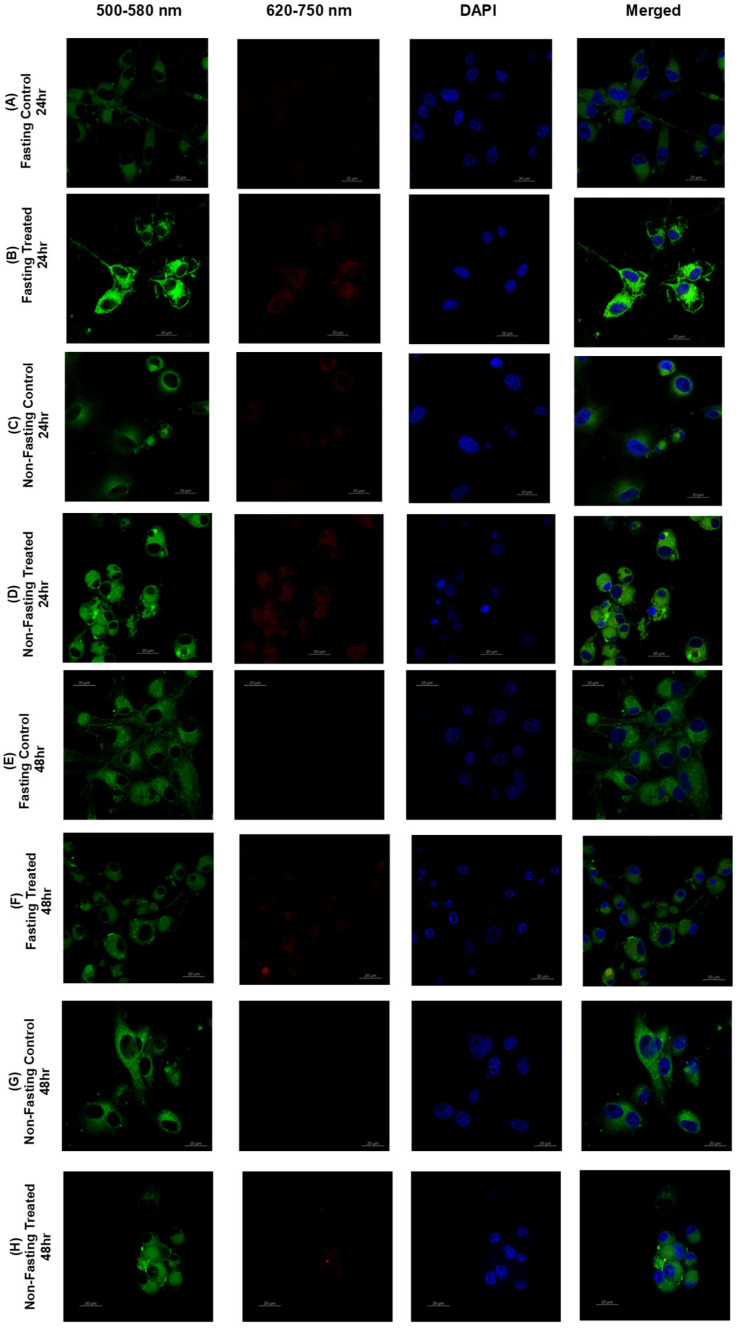
Confocal microscopic images of U-87 cells treated with atorvastatin under glucose-fasting and non-fasting conditions for 24 and 48 h. Plasma membranes stained with Di-4, nuclei stained with DAPI, green and red fluorescence indicate membrane order and disorder, respectively.

**Figure 13:**
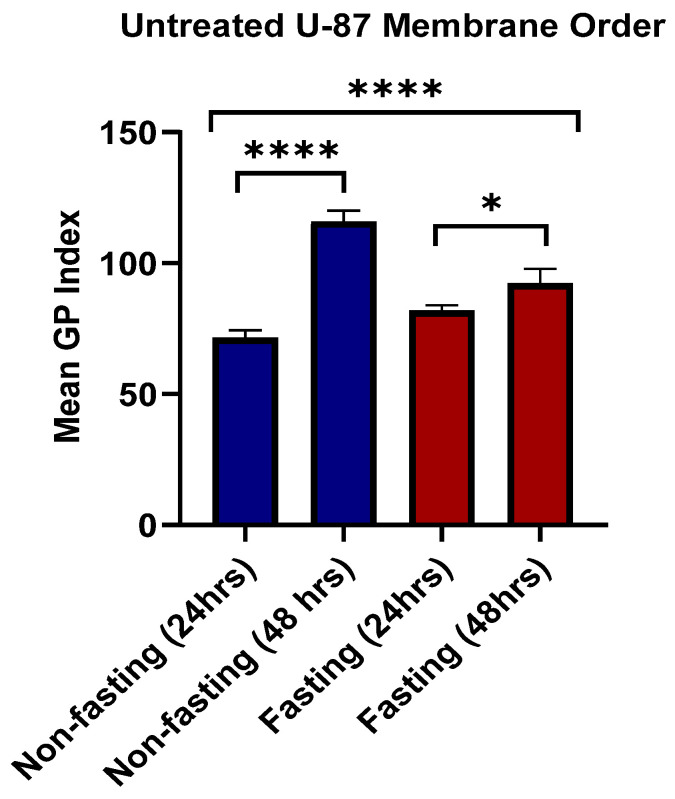
Plasma membrane order reflected by the GP index of lipid raft. One-way ANOVA indicates significantly different responses under glucose deprivation (red bars), with increased membrane order under high glucose conditions (blue bars). Data presented as mean ± SEM. Statistical significance: * *p* < 0.05; **** *p* < 0.0001.

U-87 cells demonstrated distinct responses in lipid rafts with and without glucose starvation (Figure 12). There was a significant difference in membrane order index with and without glucose deprivation (*p* < 0.0001) as shown by Figure 13. When glucose availability was high, there was a strong increase in membrane order over time (*p* < 0.0001) and a less substantial effect of time on membrane order for glucose starvation (*p* = 0.0429). These results demonstrate the significant Impact of glucose metabolism on the topology of cancer cell membranes. Glucose metabolic availability has increased membrane order at 48 h compared to the same time point of glucose deprivation. However, glucose deprivation at 24 h increased membrane order compared to glucose availability. This could be explained through the at least partially complex interactions with metabolic pathways and how these metabolic processes shape lipid composition and packing within the cell membrane over time.

**Figure 14:**
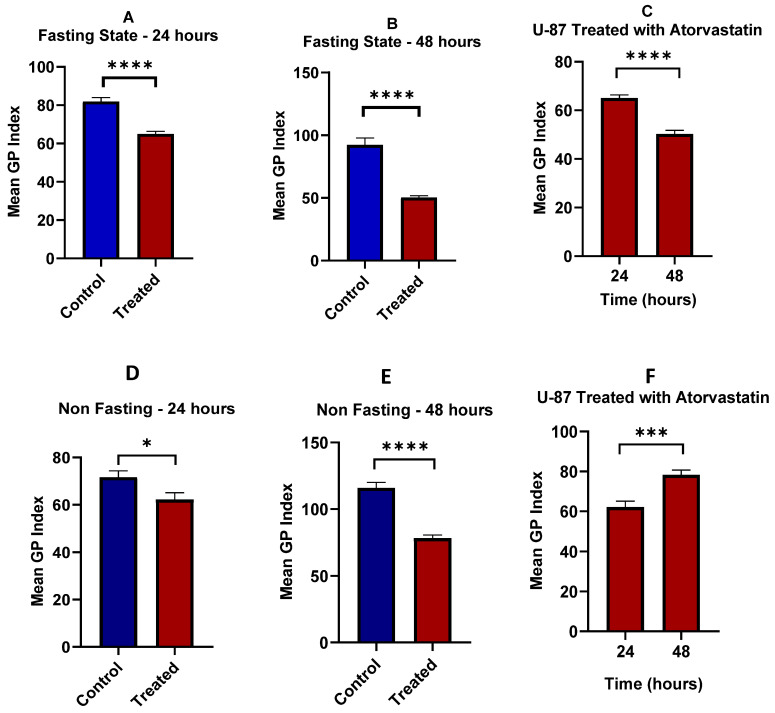
Atorvastatin treatment and fasting increase membrane fluidity in U-87 cells. (**A**) U-87 cells that have fasted for 24 h exhibit a significant reduction in the mean GP Index, indicating a decrease in membrane order and, consequently, an increase in membrane fluidity following treatment. (**B**) Fasting for 48 h results in a further decrease in the mean GP Index in treated cells compared to controls. (**C**) Atorvastatin treatment alone significantly reduced the mean GP Index over time, with a greater effect observed after 48 h compared to 24 h. (**D**) At 24 h, in non-fasting conditions, atorvastatin treatment significantly reduced the mean GP Index compared to the control group. (**E**) After 48 h of atorvastatin treatment in non-fasting cells, the mean GP Index showed a further significant decrease. (**F**) A direct comparison of the treated group in non-fasting conditions reveals a significant reduction in the mean GP Index from 24 to 48 h of atorvastatin exposure. The GP Index reflects membrane order, with lower values indicating increased fluidity. Red bars denote atorvastatin-treated cells; blue bars denote untreated control cells. Data are presented as mean ± SEM. Statistical significance: * *p* < 0.05; *** *p* < 0.001; **** *p* < 0.0001.

U-87 cells of the untreated control (Fasting Control 24 h, Figure 12A) retained their pervasive and robust green fluorescence with little to no red fluorescence. The confocal images suggest that glioblastoma cells, while fasting, are almost entirely in a highly ordered and rigid state of their plasma membrane. The approximate mean Generalized Polarization (GP) Index of 80, as demonstrated in the bar chart Figure 14A, supports this idea. In contrast, in the atorvastatin-treated (Fasting Treated 24 h, Figure 12B) cells, the fluorescence pattern was visually different. The green fluorescence is present, but it is clearly weaker than in the control cells. The red fluorescence was much more abundant, suggesting more disordered or fluid membranes. The co-localized green and red fluorescence indicates a heterogeneous landscape of membrane order, although less ordered than the untreated control. These qualitative findings are consistent with the bar chart that shows a statistically significant mean GP Index for the treated cells (*p* < 0.0001). The GP Index is a ratio of order, providing solid evidence of this fluidization effect [103].

The changes we observed align with the known mechanism of action of atorvastatin, which targets HMG-CoA and inhibits cholesterol synthesis [104]. Cholesterol plays an important role in maintaining the rigidity and shape of the cell membrane [105,106]. The findings presented here, including the reduction in the GP index and the change in fluorescence profile from green to red, provide strong support for this hypothesized mechanism, as well as the ability of atorvastatin to alter the physical properties of the U-87 cell membrane, even in a nutrient-limited (fasting) environment.

In the untreated control group (U-87 Fasting Control 48 h, Figure 12E), the cells exhibited intense, uniform green fluorescence, with no red fluorescence observed. This observation suggests that after 48 h of fasting, the U-87 glioblastoma cells remain in a highly rigid, ordered membrane state. The bar chart quantitatively confirms this finding, showing a very high Mean GP Index for the control group (Figure 14B).

In the atorvastatin-treated group (U-87 Fasting Treated 48 h, Figure 12F), the pattern of fluorescence changed substantially. The intensity of the green fluorescence was significantly less than in the control. Even more compelling, a strong red fluorescence signal was now visible throughout the cell membranes. The merged image clearly shows green and red signals. Therefore, this finding indicates there are now considerably more disordered, fluid membrane domains. This dramatic shift is quantitatively expressed in the bar chart, showing the mean GP index decreased significantly in the treated group. The highly statistically significant finding (*p* < 0.0001), establishes that once again, the effect of atorvastatin on membrane fluidity is highly reproducible and robust.

Comparison of the 48 h data with 24 h data from the previous analysis indicates a time-dependent effect. The 24 h treatment induced a shift to a more disordered membrane, but the difference seen after 48 h of treatment is even clearer. The mean GP index for the treated group decreased gradually over 24 h to 48 h, but the red fluorescence signal was brighter. A time-dependent cumulative effect of atorvastatin is occurring, resulting in a greater depletion of membrane cholesterol and greater membrane disorder. The prolonged duration of integral membrane fluidization will also produce many consequences for most biological events that depend on the membrane’s overall structural integrity and functionality, which could impact tumor cell viability and any signaling or resistance mechanisms.

The control group of cells (U87 Non-Fasting Control 24 h, Figure 12C) had bright green fluorescence with minimal to no red fluorescence, indicating that the U87 glioblastoma cell membranes are primarily highly ordered in nutrient-rich (non-fasting) conditions. The accompanying bar chart quantitatively illustrates this in the control group, where the Mean GP Index was elevated (Figure 14D).

In the atorvastatin-treated group (U87 Non-Fasting Treated 24 h, Figure 12D), we documented a completely different milieu for membrane order. The green fluorescent intensity appeared to decrease slightly, while an excess of visible red fluorescence signal was observed within the cell membranes. The merged image shown displays both green and red signals, demonstrating that the U87 cells now exhibit a higher degree of disorder. The bar chart supports this conclusion and shows that, statistically, there is a significant decrease in the mean GP index in the atorvastatin-treated cells (Figure 14D).

A significant comparison can be made between the non-fasting and fasting data. While atorvastatin treatment under both non-fasting and fasting conditions results in a significant decrease in membrane order, the extent of the change appears to be different. In fasting conditions, we observe a decrease in the mean GP Index from control to treated cells over 24 h, whereas in non-fasting conditions, we see minimal change in the GP Index over 24 h. This suggests that the baseline membrane order is higher in the fasting cells, and the atorvastatin effect, although significant in both conditions, was more pronounced in the cells experiencing the greatest metabolic stress. The ability of atorvastatin to disrupt membrane order, even when cells have sufficient nutrients, also indicates that atorvastatin has a profound effect on cellular cholesterol metabolism, which is crucial for maintaining membrane structure and homeostasis, regardless of whether the cell is under a state of metabolic stress or not.

In the untreated control group (U87 Non-Fasting Control 48 h, Figure 12G), we observed very strong, bright-green fluorescence and virtually no red fluorescence, indicating that even after a prolonged 48 h exposure to nutrient-rich media, the U87 glioblastoma cells maintained a highly ordered and rigid membrane structure. The bar graph corroborates this notion, as demonstrated by a very high Mean GP Index for the control, Figure 14E.

In the atorvastatin-treated group (U87 Non-Fasting Treated 48 h, Figure 12H), we also observed a distinct and meaningful change in the order of the membrane. The green fluorescence was still present, but its intensity had greatly reduced. A clear red fluorescence signal within the cell membranes was observed, indicating a significant increase in disordered membrane regions. This change is readily apparent in the merged image, where red fluorescence is visible and co-localized with the green signal. The accompanying bar graph illustrates that, quantitatively, the mean GP index in the control group decreased significantly compared to the treated group. The very high statistical significance (*p* < 0.0001) indicates a very robust and reproducible effect.

When the 48 h non-fasting data were compared to the 24 h non-fasting data, a clear time effect was observed again. The mean GP index for the treated group decreased for both 24 h and 48 h, and the red fluorescence signal was greater. As with the 24 h non-fasting results, atorvastatin’s modulation of membrane ordering or disorder, as shown by the fluorescence signal from the dye, was not only immediate but also continued to progress over time, producing a greater overall change in membrane properties. This supports our hypothesis that atorvastatin’s continuous inhibition of cholesterol synthesis can cumulatively deplete membrane cholesterol as the 48 h time period progresses, thereby representing an increasing membrane disorder over time. Atorvastatin was powerful enough to induce a pronounced change in the physical properties of glioblastoma cells’ membranes related to ordering, even when nutrients were abundant.

Figure 14C provides a side-by-side comparison of the Mean GP Index for fasted U87 glioblastoma cells treated with atorvastatin for 24 h. The GP Index is a measure of membrane order, and, as a result, a higher index means a more ordered, or rigid, membrane, and a lower index means a more disordered, or fluid, membrane.

The data for the treated cells is clear with respect to the time-dependent effect of atorvastatin on membrane topology. The mean GP index for the fasted cells at 24 h of treatment decreased significantly. Again, this is significantly lower than that of the respective control groups from previous analyses, confirming that atorvastatin causes membrane disorder in both types of U87 glioblastoma cells within 24 h. After treatment for 48 h, the Mean GP Index drops even more, which is even farther from the 24 h time point based on the Mean GP Index. The *p*-value indicates a statistically significant difference when compared to the 24 h time point (*p* < 0.0001).

This trend illustrates a cumulative effect of atorvastatin over time. The ongoing inhibition of HMG-CoA reductase, a direct result of atorvastatin, leads to a slow and continuous decrease in cellular cholesterol. Cholesterol is a chief regulator of membrane rigidity; the initiation of its continuing decrease causes an eventual transition of the plasma membrane from an ordered to a disordered state. Furthermore, the significant decrease in the GP index at 48 h, supported by robust statistical significance, indicates that atorvastatin does not induce membrane fluidization once it has crossed the cellular membrane; however, the ability to induce membrane fluidization as a process escalates over time. This finding provides valuable insight into the dynamics of how atorvastatin physically modifies the U87 glioblastoma cell membrane, supporting the notion that a longer treatment duration may be necessary to maximize biological utility.

Figure 14F displays a comparison of the mean GP index for non-fasting U87 glioblastoma cells treated with atorvastatin for 24 and 48 h.

The results demonstrate that the treatment has produced an unexpected and significant observation. The mean GP index at 24 h of treatment is lowered, while it rises again after 48 h of treatment. This difference is statistically significant as outlined by the *p*-value (*p* < 0.001).

This finding is roughly opposite to the results under fasting conditions, where atorvastatin treatment over time resulted in a steady decrease in the GP Index. The more ordered membrane evident in the non-fasting cells treated with atorvastatin at the 24 h and 48 h time points indicates a compensatory adjustment to the atorvastatin treatment.

One possible explanation for this divergence is that the availability of nutrients when the cells are in a non-fasting state enables the glioblastoma cells to activate cellular processes to counteract the membrane-disordering effects of atorvastatin. The main mechanism for atorvastatin is to inhibit cholesterol synthesis, but in a non-fasting state, the cells likely had the capacity to upregulate other pathways or to make other lipids or cellular structures to “make up for” the decrease in cholesterol [107,108,109]. Once compensatory responses are activated, it raises the possibility that glioblastoma cells were able to reestablish or even increase their membrane orderliness, at least after a brief period of disorder initially. These compensatory responses would not be possible if conditions were fasting, since the lack of nutrients would limit the ability of cells to mount an effective compensatory effort, and over time, the cells would progressively disorganize their membranes. This example provides a summary of the metabolic state of cells and how that state regulates clinical responses to atorvastatin.

In this study, we demonstrated a novel, pathological, time-dependent response to the drug atorvastatin on cellular membrane topology in glioblastoma cells, which was critically driven by the cells’ metabolic state. Our data depicted a clear dichotomous response by the cells depending on whether plentiful nutrients were available or not.

In the case of fasting conditions, consistent with the atorvastatin-treated cells’ inability to synthesize cholesterol, we observe a steady and consistent increase in membrane disorder across the 48 h assessment period. However, an unprecedented and unexpected finding has been made in the context of nutrient availability (non-fasting). After an initial period of membrane disorder in the treated cells, they returned to a distinctly more ordered state during the 24–48 h assessment period. This supports the notion that glioblastoma cells have an active, nutrient-driven compensatory mechanism that can effectively counteract the membrane stressors posed by atorvastatin.

**Figure 15:**
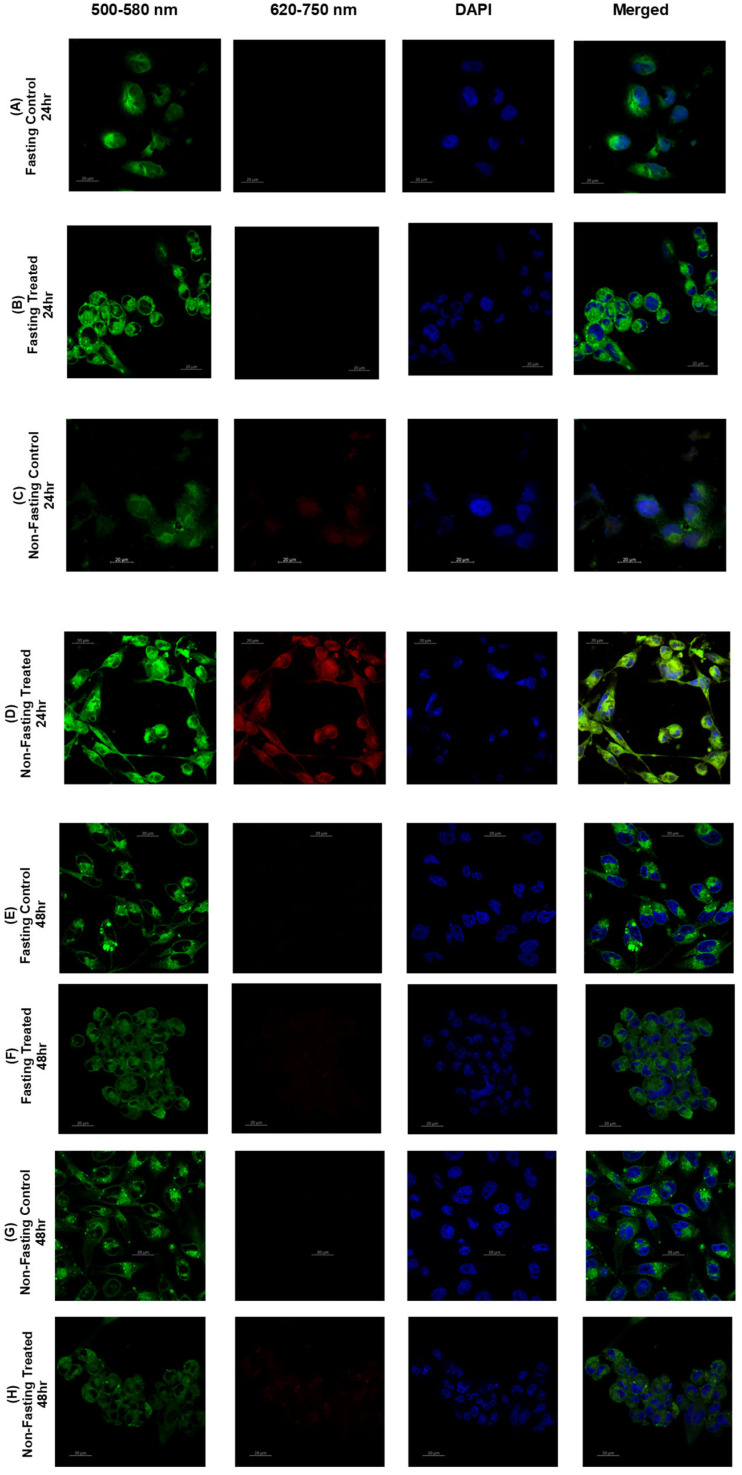
Confocal microscopic images of MDA-MB-231 cells treated with atorvastatin under glucose-fasting and non-fasting conditions for 24 and 48 h. Plasma membranes stained with Di-4, nuclei stained with DAPI, green and red fluorescence indicate membrane order and disorder, respectively.

Figure 15 shows MDA-MB-231 cells, untreated and treated samples with atorvastatin under high- and low-glucose conditions.

**Figure 16:**
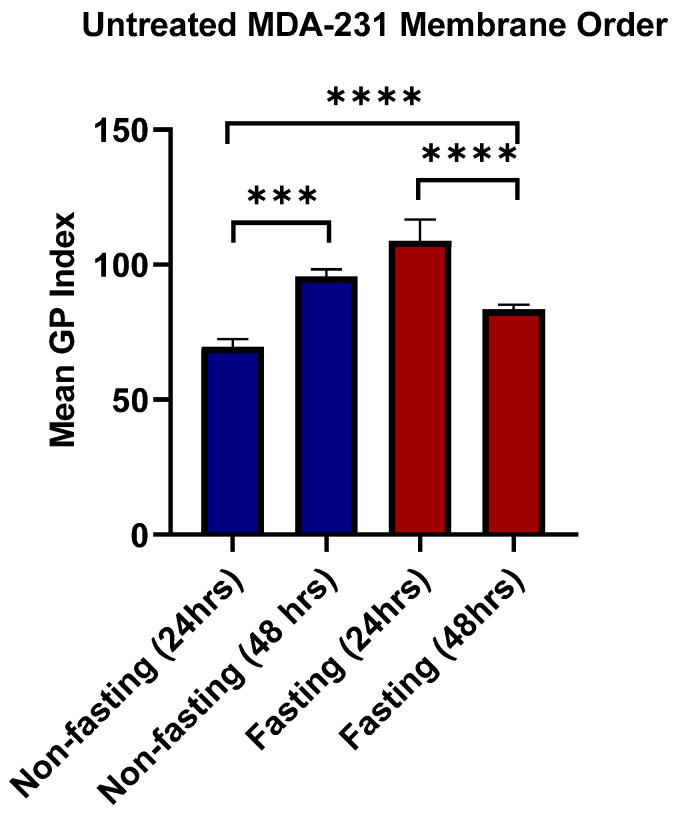
Membrane order of control samples (untreated) of MDA-MB-231 cells. One-way ANOVA reveals an increase in membrane order under high-glucose conditions (blue bars), whereas an opposite effect is observed under low-glucose conditions (red bars) (*p* < 0.0001). Statistical significance: *** *p* < 0.001; **** *p* < 0.0001.

MDA-MB-231 cells exhibited an opposite behavior in lipid rafts under glucose starvation and non-starvation conditions, with significant differences in membrane order index under the two opposing conditions (*p* < 0.0001), as indicated by Figure 16. Under high-glucose conditions, an increase in membrane order was observed over time (*p* = 0.0007). Conversely, under glucose starvation, a decrease in membrane order was observed at a later time point (*p* < 0.0001). These findings highlight the primary effects of glucose metabolism on the topology of cancer cell membranes. Glucose availability has increased membrane order. Meanwhile, glucose deprivation has decreased membrane order. This could be explained, at least in part, by the cellular requirements of glucose not only as an energy source, but also for the biosynthesis of cholesterol and lipids via the mevalonate pathway and the pentose phosphate pathway. As glucose availability is hindered, the oxidation of fatty acids and scavenging of lipids are increased, thereby enhancing lipid utilization and decreasing membrane enrichment [17,110,111,112].

**Figure 17:**
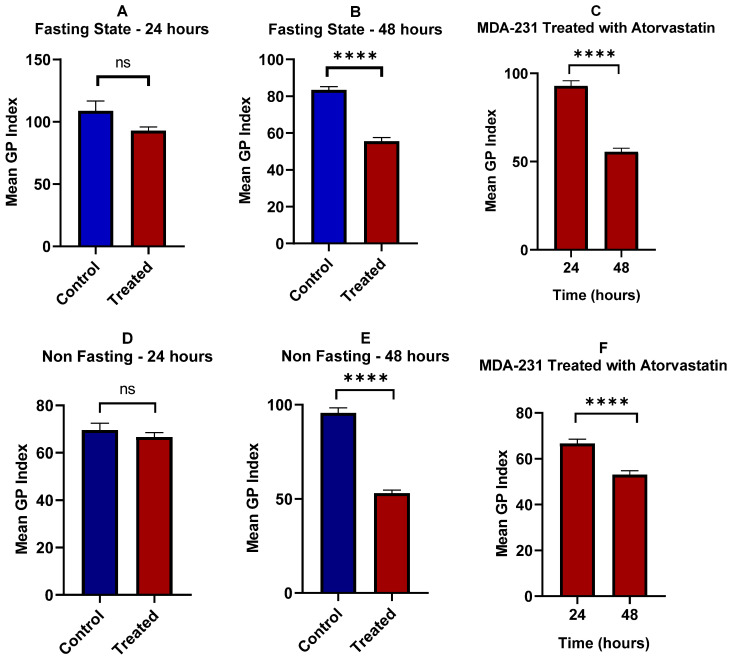
Mean GP index of MDA-MB-231 cells in response to atorvastatin under glucose-fasting and non-fasting conditions. (**A**) 24 h, glucose fasting; (**B**) 48 h, glucose fasting; (**C**) Summary comparison across glucose conditions, glucose fasting at 24 h and 48 h; (**D**) 24 h, glucose non-fasting; (**E**) 48 h, glucose non-fasting; (**F**) Summary comparison across glucose conditions, glucose non-fasting at 24 h and 48 h. Red bars denote atorvastatin-treated cells; blue bars denote untreated control cells. Data are presented as mean ± SEM. Statistical significance: ns = not significant; **** *p* < 0.0001.

Figure 17 illustrates the effects of atorvastatin on membrane order reflected by the GP index under glucose-fasting and non-fasting conditions. It has been observed that atorvastatin exerts its effects in a time-dependent manner, similar to the observations in apoptosis on MDA cells. At the fasting state and after 24 h of treatment, no significant change in membrane order was observed (*p* = 0.1049). However, after 48 h, a significant reduction in membrane order was observed (*p* < 0.0001). Comparing membrane order at the two time points (Figure 17C) shows a drastic decrease in the GP index as exposure time to Atorvastatin increases. At the non-fasting glucose state, a similar pattern was observed, with no significant change in GP index at 24 h (*p* = 0.5421), followed by a significant reduction in membrane order with prolonged exposure time (*p* < 0.0001).

Similarly to the responses of MDA-MB-231 cells to atorvastatin in terms of cellular viability and apoptosis, little dependence was observed on glucose availability, whereas more dependence was observed in response to the time factor, indicating that atorvastatin exerts its effects on membrane order in a time-dependent manner in TNBC cells. These results show that, regardless of the variability in responses among different tumor types, a similar overall pattern is observed, characterized by the effects of atorvastatin as an inhibitor of cholesterol synthesis, which decreases membrane order in both cell lines. This effect is more pronounced under glucose deprivation, especially in glioma cells.

## 4. Conclusions

This study presents novel insights into the antiproliferative effects of atorvastatin on cancer cells, highlighting its capacity to reduce cell viability, induce cell cycle arrest, initiate apoptosis to varying degrees, and disrupt plasma membrane order. The cellular response to atorvastatin was shown to be orchestrated by multiple metabolic factors, particularly glucose availability, treatment concentration, and duration of exposure. While a broadly similar pattern of atorvastatin’s effects was observed in both triple-negative breast cancer (TNBC) and glioma cells, distinct context-dependent responses emerged. Specifically, glioma cells exhibited enhanced sensitivity to atorvastatin under glucose deprivation, whereas TNBC cells demonstrated minimal glucose dependency but strong time-dependent apoptotic and membrane-disruptive effects. Notably, atorvastatin-induced cell cycle arrest was associated with marked downregulation of caspase-3 and -9, suggesting a potential involvement of caspase-mediated mechanisms. Additionally, glucose deprivation shifted the arrest phase earlier in the cycle, underscoring an enhanced effect of combining metabolic stress with antilipemic therapy. Overall, this work identifies a key adaptive mechanism in cancer cells and highlights the critical role of metabolic context in shaping therapeutic responses, a crucial consideration for the rational design of more effective, personalized cancer treatment strategies.

## Data Availability

Data is contained within the article.
